# Targeting Nucleotide Biosynthesis: A Strategy for Improving the Oncolytic Potential of DNA Viruses

**DOI:** 10.3389/fonc.2017.00229

**Published:** 2017-09-26

**Authors:** Chad R. Irwin, Mary M. Hitt, David H. Evans

**Affiliations:** ^1^Faculty of Medicine and Dentistry, Department of Medical Microbiology and Immunology, University of Alberta, Edmonton, AB, Canada; ^2^Faculty of Medicine and Dentistry, Li Ka Shing Institute of Virology, University of Alberta, Edmonton, AB, Canada; ^3^Faculty of Medicine and Dentistry, Department of Oncology, University of Alberta, Edmonton, AB, Canada

**Keywords:** adenovirus, herpes simplex virus-1, nucleotide metabolism, oncolytic virus, cancer, ribonucleotide reductase, thymidine kinase, vaccinia virus

## Abstract

The rapid growth of tumors depends upon elevated levels of dNTPs, and while dNTP concentrations are tightly regulated in normal cells, this control is often lost in transformed cells. This feature of cancer cells has been used to advantage to develop oncolytic DNA viruses. DNA viruses employ many different mechanisms to increase dNTP levels in infected cells, because the low concentration of dNTPs found in non-cycling cells can inhibit virus replication. By disrupting the virus-encoded gene(s) that normally promote dNTP biosynthesis, one can assemble oncolytic versions of these agents that replicate selectively in cancer cells. This review covers the pathways involved in dNTP production, how they are dysregulated in cancer cells, and the various approaches that have been used to exploit this biology to improve the tumor specificity of oncolytic viruses. In particular, we compare and contrast the ways that the different types of oncolytic virus candidates can directly modulate these processes. We limit our review to the large DNA viruses that naturally encode homologs of the cellular enzymes that catalyze dNTP biogenesis. Lastly, we consider how this knowledge might guide future development of oncolytic viruses.

## Introduction

Oncolytic viruses are those that preferentially replicate in, and kill, cancer cells. Most wild-type viruses are naturally oncolytic in that they generally grow best in transformed and dividing cells, but this level of specificity is rarely stringent enough for therapeutic use in cancer patients. To improve tumor specificity, researchers have modified a wide range of viruses to take advantage of dysregulated control pathways and altered signaling cascades characteristic of cancer cells. One pathway exploited in the development of oncolytic DNA viruses is that controlling the level of nucleotides available for DNA replication.

It is important that normal cells maintain the proper ratios of all four dNTPs as well as control the concentration of the dNTPs throughout the cell cycle. Otherwise they face a risk of increased mutations and genomic instability [reviewed in Ref. (1–3)]. Although the reported intracellular dNTP concentrations vary greatly depending upon the assay, cell line, and/or tissue examined, one can observe some common themes (2). Most importantly, dNTP concentrations change throughout the cell cycle in non-transformed cells, with the lowest concentration seen in resting (G0) and early G1 cells. These concentrations rise toward S-phase and then decrease as cells enter late G2 and undergo mitosis (2).

In a natural infection, viruses are most likely to encounter host cells in G0 or G1, since that is most common state of cells *in vivo*. The low level of dNTPs present at that time is a significant barrier to virus replication. Consequently viruses have evolved a number of strategies to increase the availability of dNTPs. For example, a classic strategy is one employed by small DNA viruses such as SV40, which promotes the degradation of the tumor suppressor proteins p53 and pRb, and thus drives entry into S phase. Other small viruses, including human parvovirus (4, 5) and human papilloma viruses (6), encode proteins that can cause already dividing cells to arrest at stages of the cell cycle more favorable for virus replication (i.e., S or G2). Some large DNA viruses do this as well. For example, Orf and HCMV encode proteins which disrupt the activity of the anaphase-promoting complex (APC), also leading to alterations in cell cycle progression [reviewed in Ref. (7)].

Many herpes viruses and poxviruses encode enzymes that can directly catalyze dNTP biogenesis. This is probably the most effective strategy for obtaining these critically important metabolites, as it avoids the necessity of perturbing the cell cycle, which can often trigger antiviral defenses. Numerous research groups including our own have engineered viral genomes to alter expression of viral proteins involved in dNTP synthesis in order to target virus replication specifically to tumors. Disabling the capacity of the virus to stimulate nucleotide biosynthesis in infected cells forces the virus to rely on pre-existing levels of dNTPs, which are very low in non-dividing cells but elevated in cancer cells poised for proliferation (1). A thorough understanding of nucleotide biosynthesis in normal cells and dysregulation of this pathway in infected cells and cancer should aid in the rational design of novel oncolytic viruses.

## dNTP Biogenesis and Regulation in Normal Cells

**Figure 1:**
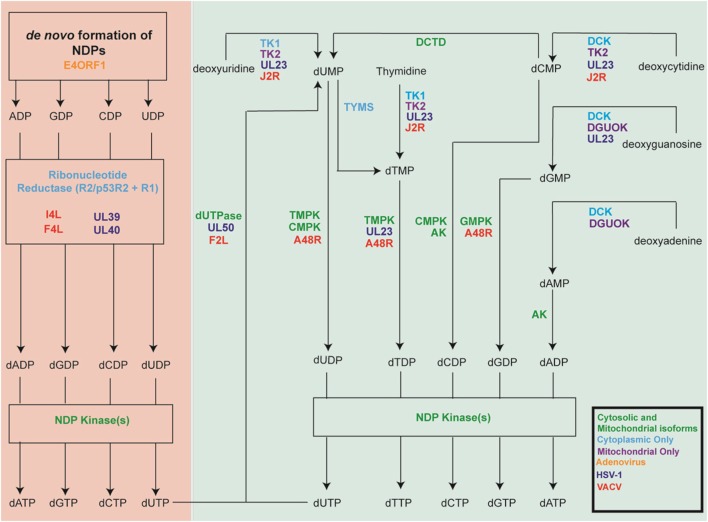
Key cellular nucleotide metabolism enzymes and their herpes simplex virus-1 (HSV-1) and vaccinia virus (VACV) homologs. Shown are the cellular enzymes that catalyze key steps in nucleotide metabolism. Green lettering indicates proteins that are expressed as both cytoplasmic and mitochondrial isoforms, while proteins found only in the cytoplasm are shown in light blue and those found only in the mitochondria are shown in purple. The figure also shows the viral genes encoding homologous proteins. HSV-1 genes are shown in dark blue, VACV in red and adenoviruse (Ad) in orange. Note that the figure over simplifies the biology because it is not possible to display all of the many different isoforms and enzymes, which sometimes also exhibit overlapping catalytic specificities.

Mammalian cells employ several mechanisms that must work in concert to provide the extra dNTPs needed for S-phase genome replication, while also maintaining the dNTP pools needed throughout the cell cycle for mitochondrial replication and for DNA repair (Table 1). At the same time, an intricate system of feedback regulation ensures that dNTP pools remain balanced. This section highlights key aspects of these pathways. Figure 1 outlines the reactions catalyzed by these cell enzymes.

**Table 1:** Key proteins, discussed in this review, catalyzing dNTP biogenesis.

Gene or protein	Natural substrate(s)	Product(s)	Comments
Ribonucleotide reductase (RNR)	ADP, CDP, GDP, UDP	dADP, dCDP, dGDP, dUDP	
R1			Protein levels remain relatively constant throughout cell cycle
R2			Cell cycle regulated, rate limiting for *de novo* nucleotide metabolism
P53-R2			Low levels throughout cell cycle, induced in response to DNA damage
NMP kinases			
Thymidylate kinase (TMPK)	dTMP, dUMP	dTDP, dUDP	Mitochondrial and cytoplasmic isoforms exist
Cytidine/uridine monophosphate kinase (CMPK)	CMP, dCMP, UMP, dUMP	CDP, dCDP, UDP, dUDP	Mitochondrial and cytoplasmic isoforms exist
Guanylate kinase (GMPK)	GMP, dGMP	GDP, dGDP	Mitochondrial and cytoplasmic isoforms exist
Adenylate kinase (AK)	AMP, dAMP, CMP, dCMP	ADP, dADP, CDP, dCDP	Multiple isoforms and tissue-specific species exist
NDP kinases	NDPs, dNDPs	NTPs, dNTPs	
Thymidine kinase (TK)			
TK1	dT, dU	dTMP, dUMP	Cytoplasmic, cell cycle regulated
TK2	dT, dU, dC	dTMP, dUMP, dCMP	Mitochondrial localization, expressed at low levels throughout cell cycle
dCMP deaminase (DCTD)	dCMP	dUMP	
dUTPase	dUTP	dUMP	Mitochondrial and cytoplasmic isoforms exist
Thymidylate synthetase (TYMS)	dUMP	dTMP	Cytoplasmic. Unclear if mitochondrial isoform exists
Deoxycytidine kinase (DCK)	dA, dG, dC	dAMP, dGMP, dCMP	Cytoplasmic only
Deoxyguanosine kinase (DGUOK)	dA, dG	dAMP, dGMP	Mitochondrial only

Three of the four cellular dNTPs (dATP, dGTP, and dCTP) are products of pathways largely dependent upon the activity of ribonucleotide reductase (RNR). Several types of RNRs exist. Mammalian RNRs are class I enzymes, comprising a heterotetramer consisting of two large subunits (R1 or RRM1) and two small subunits (R2 or RRM2) [reviewed in Ref. (8)]. RNR reduces NDPs (ADP, GDP, CDP, and UDP) to their respective dNDP forms, which are subsequently converted to dNTPs by cellular NDP kinases. RNR regulation is complex and involves an intricate system of feedback inhibition which allows RNRs to sense specific dNTP levels and alter the rate of reduction of other NDPs so as to maintain balanced dNTP pools [interested readers are invited to read (8)].

Cellular RNR protein levels are regulated by a combination of transcription and posttranslational processes. Few R1 or R2 mRNA transcripts are found in G0 cells, but these then rise as cells enter G1 and S-phase. The R1 protein exhibits a long half-life (~20 h), thus R1 protein levels remain relatively constant throughout the cell cycle. In contrast, the R2 protein is much less stable with a half-life of only ~3 hr. As the demand for dNTPs declines at the end of S-phase, the R2 protein is targeted for degradation by the Skp1/Cullin/F-box complex during G2, and by Cdh1-APC during mitosis. These reactions are promoted by interactions with the “KEN box” domain found near the R2 N-terminus (9). Because of these processes, the levels of the R2 subunits are thought to rate-limit the formation and stability, and therefore activity, of the RNR complex.

Mammalian cells also encode a second R2 species called p53R2 (or RRM2b). R1 can complex with p53R2, and although active, this RNR isoform exhibits lower catalytic activity than RNRs composed of R1 and R2 (10, 11). The p53R2 protein is expressed throughout the cell cycle and its presence is thought to ensure that sufficient dNTPs are available to support mitochondrial genome replication outside of S-phase (11, 12). As the name implies, p53R2 expression can also be upregulated in a p53-dependent manner in response to DNA damage (13). This is thought to increase the availability of the dNTPs needed for DNA repair.

Although *de novo* synthesis from NDPs is thought to be the major pathway responsible for dCTP, dGTP, and dATP production for nuclear DNA synthesis, salvage enzymes exist that can also contribute to their biosynthesis. This includes deoxycytidine kinase (DCK), which is not cell cycle regulated and can phosphorylate dC, dG, and dA (14). The resulting dCMP, dGMP, and dAMP can be further phosphorylated to dNDPs by a variety of nucleoside monophosphate kinases including the many adenylate kinases, uridylate-cytidylate kinases (UMP-CMPK), and guanylate kinases (GMPK) reviewed in Ref. (15). The resulting dNDPs are converted to triphosphates by the same NDP kinases used for *de novo* synthesis of dNTPs.

Cells do not have a source of rTDP that could serve as an RNR substrate for dTDP production and so other enzymatic routes are used to manufacture dTTP (Figure 1). The production of dTTP requires thymidylate kinase (TMPK), which converts dTMP to dTDP. NDP kinases then convert dTDP into dTTP. There are two sources of dTMP in mammalian cells. One route employs thymidylate synthetase (TYMS) to convert dUMP into dTMP. Most of this dUMP is thought to come from deamination of dCMP by dCMP deaminase (DCTD) (16) although dUMP can also be produced from the pyrophosphorolysis of dUTP by dUTPase. The latter route has the dual function of reducing the incorporation of uracil into DNA, by DNA polymerases, which if not repaired by uracil N-glycosylases (UNG, also designated UDG) can be mutagenic. The second source of dTMP is through thymidine kinase (TK) catalyzed phosphorylation of thymidine. Cells express both cytoplasmic (TK1) and mitochondrial (TK2) forms of the enzyme (17). Both forms can convert thymidine to dTMP and deoxyuridine to dUMP, while TK2 can also convert deoxycytidine to dCMP (17). The availability of thymidine is influenced by the activity of thymidine phosphorylase (TYMP). Although the enzyme can interconvert thymine and thymidine, it enzymatically favors the catabolism of thymidine (18).

Enzymes catalyzing dTTP formation can be cell cycle regulated in a variety of ways. The E2F-pRb pathway that drives entry into S phase has been shown to regulate the transcription of TYMS, dUTPase, TK1, and TMPK (19–22). TK1 activity is also subject to additional levels of regulation, with its transcription and translation efficiency also promoted by p107 and cyclinA/Cdk-2 (22). After DNA replication, TK1 activity is reduced following phosphorylation by Chk1 kinase (23, 24). Furthermore, TK1, TMPK, and TYMS are degraded at the end of mitosis, in a process that is mediated by APC (25, 26).

Balancing these biosynthetic pathways, are enzymes that catabolize unwanted dNTPs, including pyrophosphatases like the dUTPases. These may help maintain balanced dNTP pools and reduce the availability to virus replication machinery. Recently, a dCTPase, DCTPP1, was identified in human cells, which is thought to regulate and sanitize dNTP pools by degrading dCTP or dCTP analogs such as 5-methyl-dCTP (27). DCTPP1 levels vary with the cell cycle, but in a manner opposite to the behavior of anabolic enzymes (27). Another enzyme that catabolizes dNTPs is SAMHD1 (28, 29). In addition to being a ribonuclease, SAMHD1 has a dNTP triphosphorylase activity. In the presence of dGTP, SAMHD1 hydrolyzes dNTPs releasing a deoxyribonucleoside and triphosphate. The enzyme is subject to complex regulatory schemes that differ in different cell types.

Mitochondrial DNA replication is not subject to cell cycle control, so there must be mechanisms to maintain mitochondrial dNTP pools that are separate from those used for nuclear DNA synthesis [reviewed in Ref. (2, 30)]. This is done through two mechanisms. First, transport proteins can move dNMPs, dNDPs, or dNTPs from the cytoplasm of cycling cells into the mitochondria, where mitochondrial NMPK/NDMKs can generate dNTPs. Cytoplasmic and mitochondrial dNTP pools appear to mix in dividing cells (31), and while dNDPs and dNTPs cannot passively diffuse across mitochondrial membranes, some nucleotide transport proteins are capable of exchanging nucleotides across the membrane (32). Alternatively, in non-dividing cells, mitochondria are thought to mostly generate dNTPs using mitochondrial enzymes and salvage pathways, while in the cytoplasm catabolic reactions degrade dTMP to thymine. Besides the aforementioned TK2 which is encoded by a separate gene from TK1, mitochondrial isoforms of TMPK (33), dUTPase (34), and UNG (35) have been identified. Unique to the mitochondria is deoxyguanosine kinase (DGUOK), which converts dG and dA to dGMP and dAMP, respectively (36). While no evidence of mitochondrial TYMS activity has been reported, some groups have detected mitochondrial RNR activity suggesting that *de novo* dNTP synthesis may also be possible (30).

The differences between the mechanisms of cytoplasmic and mitochondrial dNTP biogenesis may contribute to the differences in the relative abundance of each nucleotide. For example, while dGTP is the least abundant nucleotide in the cytoplasm, it is the most abundant in mitochondria (37). Mutations in genes required for nucleotide biosynthesis (e.g., p53R2, TK2, and DGUOK) produce defects in mitochondrial DNA replication and have been linked to several inherited human diseases (38–40).

## Altered dNTP Biogenesis in Cancer

**Figure 2:**
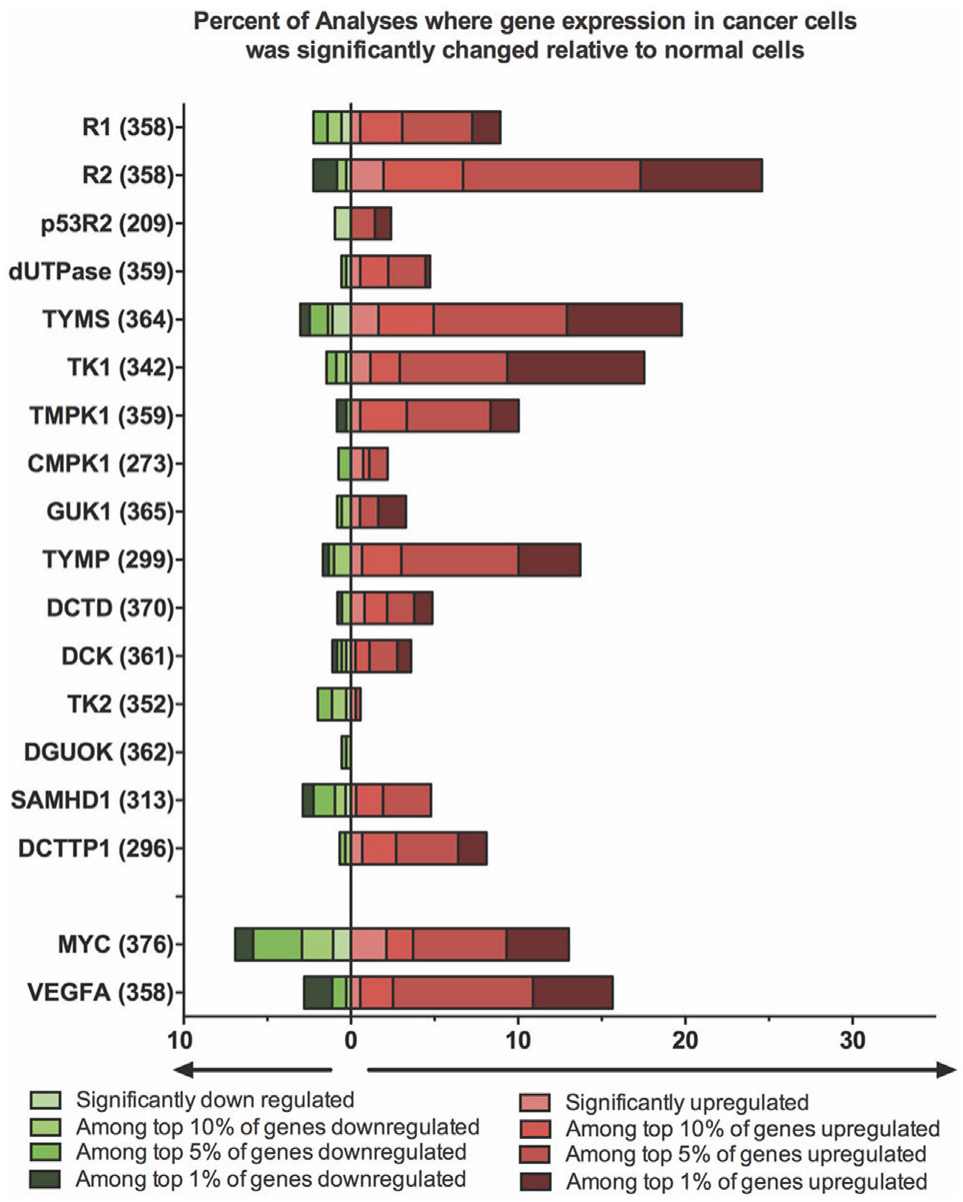
Expression of deoxynucleotide biosynthesis genes is altered in a significant portion of cancer cells. A differential analysis of mRNA expression between cancer and normal tissue was performed for each gene using Oncomine Research Edition (Version 4.5) software. A change in mRNA expression greater than twofold, with a *P*-value <10^−4^, was considered significant. The percent of analyses where gene expression was significantly changed in cancer cells was then calculated and plotted. The fraction of samples where gene expression was among the top 1, 5 or 10% of genes whose expression was altered was also calculated. The total number of analyses surveyed in the data, are denoted in brackets beside each gene. Data were retrieved and analyzed on April 6, 2017. Two genes (Myc and VEGFA) whose expression is frequently altered in cancer cells are included for comparison.

A number of excellent reviews have summarized what is known about the links between altered dNTP metabolism and its relationship to cellular transformation and cancer (2, 29, 41). Here, we summarize some of the key points relating to how the genes and proteins outlined above influence proliferation of cancer cells, how these systems can be targeted by anticancer therapies, and how alterations in nucleotide metabolism can affect the outcome of cancer treatments. Lastly, we provide a comprehensive analysis of the changes in relevant gene expression patterns that are seen in many different cancer types, using the Oncomine database (Figure 2). These changes provide insights into how one might align the properties of an oncolytic virus with the cancer it targets.

The best-known and most exhaustively studied links between nucleotide metabolism and cancer concern RNR, in part because the enzyme is a target of many widely used chemotherapeutic agents. Mutations in both R2 and p53R2 have also been linked to tumorigenesis, while R1’s role is less clear and R1 has been reported to function as a tumor suppressor (41). These seemingly contradictory roles reflect the different ways that RNR activity can affect normal and transformed cell processes, and the differing perspectives clinicians and researchers apply to studying this enzyme in health and disease. As a general rule, any increase in dNTP biosynthesis is expected to favor tumor growth and also decrease the therapeutic effectiveness of a nucleotide analog that competes with these dNTPs. As noted above, the fact that the R1 and R2 subunits are differentially regulated through the cell cycle adds additional complexity to interpreting the links between RNR and cancer.

Although the mechanism is not clear, R1 overexpression has been shown to induce PTEN expression and to reduce transformation and tumorigenesis in animal models (42–44). Furthermore, R1 overexpression correlated with better survival in patients with non-small cell lung carcinoma following surgery alone (45). However, R1’s role in supporting DNA repair has also been suggested to explain why elevated R1 expression is linked to increased resistance to platinum drugs and gemcitabine (whose target includes RNR), and has been suggested to be used as a biomarker for tailoring an individual’s cancer treatment plan to these drugs [reviewed in Ref. (41)].

In contrast to R1, R2 dysregulation is clearly associated with oncogenesis. Increased R2 expression is linked to increased DNA replication, and this is associated with greater uracil incorporation leading to more DNA breakage and mutation (46, 47). Elevated R2 levels have also been associated with increased cell proliferation, invasiveness and tumor vascularization (41, 48–50). The role of p53R2 is less clear. It is induced in response to signaling through p53 and increased p53R2 levels correlated with better prognosis in some cancer patients (41). However, like R2, p53R2 overexpression also increased the rate of spontaneous tumor formation in transgenic mice (47). Using the Oncomine database, Aye et al. found that a significant fraction of cancer specimens exhibited increased R1 and R2 levels, but alterations in expression of p53R2 were less common (41). Our own analysis supports these earlier findings (Figure 2).

The enzymes involved in dTTP biosynthesis (TYMP, TYMS, TK1, and TMPK) are also upregulated in transformed cells facilitating increased rates of DNA synthesis, and several chemotherapeutic strategies target these pathways (19, 51, 52). Fluoropyrimidines like 5′-fluorouracil (5-FU) are activated by TYMP and TK1 and then bind to and inhibit TYMS. The active metabolites can also serve as DNA polymerase α substrates and are then incorporated into DNA (53). Antifolates, like methotrexate, also reduce dTTP production by blocking production of the co-factors used by TYMS. Upregulation of TYMP, that interconverts thymine and thymidine, promotes tumorigenesis, angiogenesis, and metastasis and has been implicated in suppressing innate immune response in tumors and perturbing energy metabolism [reviewed in Ref. (18)]. At the same time, increased TYMP levels can enhance sensitivity to some fluoropyrimidines (18). Although chemotherapeutic agents targeting these pathways may initially be effective, the high mutation rate in cancer cells coupled with strong selective pressure can lead to the development of resistance. For example, 5-FU treatment can drive selection for increased TYMS levels, leading to 5-FU resistance and poorer prognosis (19, 51, 54). A combination of metformin and 5-FU treatment can also induce 5-FU resistance, although this creates gemcitabine sensitivity probably due to a concurrent increase in DCK activity (55).

TK1 levels are elevated in cells dysregulated in several pathways commonly associated with cancer, including the E2F-pRb pathway. A classic example of dysregulation of this pathway is that induced by the SV40 large T antigen that binds to and inactivates pRb (56). SV-40-transformed TK-deficient cells were less able to form tumors in hamsters, compared to those with a functional TK, suggesting that TK also promotes tumorigenesis (57). Furthermore, it has recently been shown that upon re-entering the cell cycle, terminally differentiated muscle cells did not express sufficient TK1 activity to support efficient proliferation, suggesting that low levels of TK1 may play a role in preventing aberrant replication (58). At the same time, TK1-dependent nucleotide biosynthesis is required for DNA repair. Increased TK1 activity can enhance the excision of genotoxic damage and thus promote resistance to many cancer therapies (59). TK levels are elevated in many cancer cells, regardless of the cell’s proliferation state (60), and since this can sometimes be detected in serum it has been suggested that TK might be used as a prognostic marker (61, 62). TK activity also increases the efficacy of fluoropyrimidines (54).

Thymidylate kinase activity can reduce the amount of dUTP incorporated into DNA during repair by providing sufficiently high levels of dTTP at the damaged site (61). It has been suggested that this may reduce the risk of double-stranded breaks produced by UNG-mediated DNA repair. In agreement with this, inhibiting TMPK sensitizes cells to the DNA damaging agent doxorubicin, and treatment with this combination produced superior tumor control (63). Cancer cells have also been shown to exhibit altered levels of other monophosphate kinases (CMPK1, GMPK) and this sometimes correlates with differences in cancer prognosis and the response to some chemotherapeutics (64–66).

Deoxycytidine kinase phosphorylation of the chemotherapeutic agents AraC and gemcitabine is required for their inhibition of DNA polymerase, and the loss of DCK activity has been linked to AraC and gemcitabine resistance (67–69). An analysis of pancreatic cancer patients treated with gemcitabine found that DCK levels correlated with prolonged survival (70, 71), but little else has been reported linking DCK to cancer.

Altered expression or mutation of dNTPase enzymes have been seen in several cancers. SAMHD1 has been suggested to have tumor suppressor activities, as its overexpression can decrease cellular proliferation, and mutation, and loss of SAMHD1 has been reported for several cancers including leukemias, lymphomas, breast, and colorectal cancer (29, 72). SAMHD1 levels also differentially affect common chemotherapeutics. SAMHD1 can contribute to AraC resistance, with lower levels of SAMHD1 correlating with increased AraC sensitivity (73). Furthermore, a retrospective analysis of AraC-treated AML patients found that individuals with lower SAMHD1 levels experienced better outcomes (73). While decreased SAMHD1 levels increase AraC sensitivity, SAMHD1 overexpression increases sensitivity to other DNA-damaging agents such as etoposide, mitomycin C, or camptothecin (72). These observations can be explained by the effects SAMHD1 has on dNTP pools. High levels of enzyme activity would decrease dNTP concentrations, thus slowing cell proliferation, decreasing rates of error-prone DNA replication, and inhibiting repair of lesions caused by direct acting DNA damaging agents. Conversely loss of the enzyme would promote dNTP-dependent replication and cell proliferation. The way SAMHD1 activity modulates AraC sensitivity is explained by the recent observation that it catabolizes a variety of nucleoside analogs (74).

Both DCTPP1 and dUTPase have been linked to tumor invasiveness. DCTPP1 expression has been reported to be elevated in breast and gastric cancers, and these expression levels correlated with poorer prognosis (75, 76). The expression of dUTPase is regulated by p53, and elevated in p53-mutated cells (21). In Huh7 cells, siRNA-mediated silencing of dUTPase decreased both invasiveness and cell growth, suggesting that dUTPase levels may affect tumor establishment or progression (77). However, dUTPase activity also seems to be important in the context of R2 levels. Decreased dUTPase levels increase the rates R2-induced genomic instability. A study examining the survival of colorectal cancer patients revealed that patients with high R2 and low dUTPase levels faced a poorer prognosis than patients with elevated levels of both R2 and dUTPase (46), perhaps due to the enhanced flux of dUTP that would be produced relative to dTTP formed by TMPK (63). Both DCTPP1 and dUTPase contribute to cellular resistance to the chemotherapeutics 5-FU and decitabine (76–78). 5-FdUTP is a known substrate for cellular dUTPases, suggesting an obvious mechanism of action (79).

Using publicly available data sets in the Oncomine database, we have examined changes in expression of genes involved in nucleotide metabolism in different tumor types compared to normal tissues (Figure 2). The microarray data searched on April 6, 2017, comprised 19 major cancer types (plus an “other category”) and surveyed ~350 unique tests per gene. It is clear from these analyses that it is very common for genes involved in dNTP synthesis to be highly upregulated in cancer. In particular, TK1, R2, and TYMS mRNAs were found more frequently than *MYC* among the top 1% of overexpressed genes. The same conclusions are supported by histochemical protein analyses and tissue microarray data posted by the Human Protein Atlas (http://www.proteinatlas.org). A search of these data (v.17) showed that the TK1, R2, and TYMS proteins are expressed at high or medium levels in 25, 79, and 83% of the 20 cancer types surveyed, respectively. The high frequency with which a protein like R2 is overexpressed in so many cancer types highlights the tumor specificity that could be afforded by exploiting this pathway when designing oncolytic viruses.

## Adenovirus (Ads)

Adenoviruses are non-enveloped double-stranded DNA viruses that replicate in the nucleus of infected cells. They have been widely tested as gene therapy vectors and oncolytic agents and an early region 1b (E1b)-deleted Ad (H101/Oncocrine) was licensed in China in 2006 for use in treating head and neck cancer. While numerous strategies for engineering Ad-tumor specificity have been described [reviewed in Ref. (80, 81)], this section will focus on how Ads alter dNTP levels, and how this might be used as way of generating oncolytic variants.

Wild-type Ad infection has been reported to increase dNTP levels and its growth is influenced by dNTP availability (82, 83). However, Ads do not encode homologs of the cellular enzymes that catalyze nucleotide biosynthesis; therefore, they likely increase dNTP levels by indirect methods. For example, Ads encoding *E4-ORF1* mutations grow more poorly than wild-type virus, and cells infected with such mutants exhibit reduced dNTP levels compared to wild-type virus infected cells (83). Although E4-ORF1 shows some resemblance to the dUTPase genes encoded by humans, herpes simplex virus-1 (HSV-1) and VACV, the purified Ad protein lacks dUTPase activity (84). Instead, E4-ORF1 seems to indirectly increase dNTP levels by activating the MYC and AKT signaling pathways that upregulate enzymes involved in the formation of ribose (83). When combined with mutations in *E1b*, a virus bearing an *E4-ORF1* deletion showed an increased ability to kill cancer cells in culture (85). Whether *E4-ORF1* mutant viruses might have independent oncolytic properties, or could improve the efficacy of other existing oncolytic Ad strains *in vivo*, is unknown.

A ΔE1ΔE3 Ad expressing human R1 exhibited a superior ability to control the growth of human colon adenocarcinoma tumors in CD-1 nude mice following intratumoral virus administration of Ref. (86). Since this is a non-replicating virus the effect would have to be attributed to R1’s other tumor-suppressor activities.

## Vaccinia Virus

Poxviruses are large double-stranded DNA viruses which replicate in the cytoplasm of infected cells. While capable of attaching to and entering a wide variety of cells, postentry factors are thought to define cellular and host tropism (87). A number of poxviruses have been tested for their potential as oncolytic agents (Table 2), but the two in the furthest state of development are the *Leporipoxvirus* myxoma virus (MYXV) and the *Orthopoxvirus* vaccinia virus (VACV). MYXV infects rabbits and hares, but naturally has oncolytic properties in some cancers. It is currently being developed for use in the treatment of hematological cancers (88–90).

**Table 2:** Oncolytic vaccinia viruses with mutations in nucleotide metabolism genes.

Virus	Strain	Mutation(s)	Transgene(s)	Status	Reference
JX-594^a^	Wyeth (NYCBOH)	TK^−^	GM-CSF *lacZ*	Currently being evaluated in a phase III trial for HCC	(103, 107, 115, 118–120, 123, 206, 262–267)
vvDD-CDSR (JX-929)^b^	Western reserve	TK^−^, VGF^−^	Yeast cytosine deaminase, *lacZ*, human somatotropin receptor 2	Completed phase I trial	(105, 109, 127–129, 133, 268)
GL-ONC1 (GLV-1h68)^c^	Lister	TK^−^, *A56R*^−^, *F14.5L*^−^	*Renilla* luciferase-GFP, *lacZ, gusA*	Currently in phase I/II trial	(134–136, 138, 269–271)
VV-FCU1	Copenhagen	TK^−^	FCU1	Preclinical	(114)
VV-FCU1	Western reserve	TK^−^, R1^−^	FCU1	Preclinical	(157)
ΔF4L	Western reserve	R2^−^	Neomycin, *gusA*, and mCherry	Preclinical	(104, 150)
ΔF4LΔJ2R	Western reserve	TK^−^, R2^−^	*lacZ*, neomycin, *gusA*, and mCherry	Preclinical	(104, 150)
TK^−^/ΔJ2R^d^	Various	TK^−^	Various	Preclinical	

*^a^The JX-594 backbone of contains a natural truncation in the B18R gene not found in other recombinant VACVs (116)*.

*^b^While VVDD-CDSR is the lead candidate and only version of a TK^−^/VGF^−^ VACV in clinical trial, other viruses that contain the same mutations, but have additional modifications exist. Some examples include a version that expresses GFP or luciferase, a version (JX-963) that expresses GM-CSF *in lieu* of CDSR, and a version (VVDD-A34R_K151E_) which encodes a mutation that enhances viral spread throughout tumors (91, 129, 130, 268, 272)*.

*^c^While GL-ONC1 is the lead candidate, and only version of a TK^−^/A56R^−^/F14.5L^−^ VACV in clinical trials, other modifications to this backbone have been made and are being tested in preclinical settings. Some examples include versions which express MMP-9, Cdc6, hEPO, or hNIS [reviewed in Ref. (139)]*.

*^d^The TK (J2R) loci is disrupted in a number of recombinant VACVs and has been used as a comparator for evaluating many other recombinant VACVs*.

Vaccinia virus has a well-established safety record based on its extensive use as a smallpox vaccine. While VACV naturally displays preferential growth in cancer cells (91), it can productively infect a wide variety of animals and cell types, including non-dividing cells. This is facilitated by a combination of specific host range factors as well as genes that support general replication in a variety of cell types. Such proteins are attractive targets for improving the oncolytic selectivity of the virus. Some examples include immunoregulatory proteins, viral growth factors (VGFs), and the subject of this review—enzymes involved in nucleotide metabolism.

The repertoire of nucleotide metabolism genes encoded by poxviruses varies by genus. VACV and other orthopoxviruses encode the largest array of these genes including catalytically active homologs of R1, R2, TK, TMPK, dUTPase, and UNG. Some VACV strains also encode a GMPK, although whether this is functional, and contributes to GDP production, is unknown (92).

### VACV TK and TMPK Mutants

Vaccinia virus encodes two separate enzymes that catalyze biosynthesis of dTTP—a TK homolog encoded by the *J2R* gene, and a TMPK homolog encoded by the *A48R* gene. Like mammalian TK1, VACV TK is a class II enzyme, forms a homotetramer, and phosphorylates thymidine (93–95). It is also inhibited by dTTP and can phosphorylate thymidine analogs such as AZT, AraT, and BrdU (95, 96). Unlike mammalian TK1, VACV TK has a limited capacity to phosphorylate deoxycytidine (95). Mammalian cells can become BrdU-resistant through the loss of TK activity (97), and the availability of TK^−^ cells led to the discovery that VACV must encode a TK activity (96) and also permitted the isolation of BrdU-resistant/TK-deficient viruses (98). This method facilitated the identification of the gene responsible for TK activity in the early 1980s (99–101) and led to the widespread use of the TK locus as a site for incorporating transgenes. Compared to cells infected with wild-type VACV, those infected with TK mutants show decreased levels of dTTP and dCTP (102).

Cellular TK activity can complement mutations in viral TK and provides a basis for producing tumor-specificity. This is best highlighted by studies using shRNA- or siRNA-mediated silencing of cellular TK in HeLa cervical carcinoma cells. Although wild-type and TK^−^ viruses grow equally well in HeLa cells under ordinary conditions, when cellular TK is silenced the growth of a TK mutant (JX-594) is reduced about fivefold, whereas the wild-type virus is unaffected (103). Serum-starvation has also been reported to decrease the levels of cellular TK in normal cells, but to a lesser extent in cancer cells (91). While cancer cells cultured under normal or serum-starved conditions support equal growth of both TK-mutant and wild-type virus, one sees decreased transcription and yields of TK mutants relative to wild type in many untransformed cells cultured under serum-starved conditions (91, 104). It should be noted that this differential virus growth is not seen with all serum-starved normal cell lines (104), which could be due to the fact that dTTP can also be generated in a TK-independent manner, through TYMS. The selective replication of TK mutants in cancerous tissue has also been shown in animal models (105, 106), in explanted patient tumors, and in biopsies taken from patients receiving virus as part of a clinical trial (107).

TK mutants are considerably less virulent than wild-type VACV in animal models; however, the degree of attenuation depends upon the administration route and genetics of both virus and host. In a mouse BALB/c intracranial infection model, TK mutations increased the LD_50_’s ~25-fold using a VACV Wyeth strain (3.2 × 10^6^ versus 9.1 × 10^7^ PFU) and ~4,000-fold higher using a Western Reserve (WR) strain (10 versus 4 × 10^4^ PFU) (108). TK mutants also showed decreased pathogenesis in rhesus macaques, as evident by decreases in the size of necrotic lesions after intradermal administration (109). However, in immune compromised animal models, TK mutants can still be virulent. McCart et al. compared the pathogenesis of wild type and TK mutants in a WR strain background following intraperitoneal administration to nude mice. They found that while wild-type virus killed the mice by day 5, mice injected with TK mutants survived to day 17 (105). The TK mutants showed a ~100-fold reduction in titer relative to wild-type virus in the brain, but differences were insignificant in other tissues including spleen, ovaries, testes, and bone marrow (105). Our laboratory has also examined the pathogenicity of a TK mutant in tumor-bearing immune compromised animals at doses sufficient for oncolytic activity and found significant virus titers in normal tissues, particularly ovaries, as well (104).

In general, while TK mutations promote increased safety and selectivity and are the basis of all the VACV oncolytics that have entered the clinic, TK deletions have always been combined with mutations in other viral genes and/or transgenes. TK mutants exhibit increased safety and/or efficacy when combined with mutations in the VGF (105), R1 or R2 (104, 110), or the type I interferon binding protein (*B18R*). TK has also served as a site for integration of transgenes encoding granulocyte-macrophage colony-stimulating factor (GM-CSF, described below), hydroxyprostaglandin dehydrogenase (111), the pattern recognition receptor DNA-dependent activator of IFN-regulatory factors (DAI) (112, 113), TIR domain-containing adaptor inducing IFN-β (TRIF) (112), recombinant antibodies such as those targeting PD-1 (110), a fusion of yeast cytosine deaminase and uracil phosphoribosyltransferase (FCU1) (114), and many antigens used in recombinant vaccines.

JX-594 (Pexastimogene devacirepvec or Pexa-Vec) is the oncolytic VACV in the furthest state of clinical development. This Wyeth strain of TK^−^ virus also encodes human granulocyte-macrophage colony-stimulating factor (GM-CSF) and beta-galactosidase (115). The *B18R* gene in this recombinant also naturally encodes a truncated protein (116), which is also known to reduce virulence (117). GM-CSF has many functions including the ability to stimulate the development of hematopoietic cells and has been shown to improve the efficacy of a TK^−^ virus in rabbits bearing VX-2 liver tumors (91).

JX-594 is being developed by Silagen and has been tested in at least 11 clinical trials. Over 300 patients with a wide variety of cancers including melanomas, and hepatocellular, colon, pancreatic, lung, and ovarian carcinomas have been enrolled, and the virus has been administered by both intratumoral and intravenous routes (103, 107, 115, 118–124). JX-594 is generally well tolerated, with most patients experiencing transient flu-like symptoms. Maximum tolerable doses of 10^9^ PFU have been reported when administered intratumorally (124), while a maximum feasible dose of 10^9^ PFU has been reported for intravenous administration (107, 119). A phase I trial in seven children with stage III or IV cancers showed similar safety profiles to that seen with adults (121). Evidence of tumor-specific replication, transgene expression, development of virus neutralizing antibodies, and efficacy have been observed in subsets of patients in these studies.

In a phase II dose-finding study of JX-594 in 30 patients with advanced hepatocellular carcinoma (HCC), patients receiving the highest dose of virus (10^9^ PFU) exhibited longer survival than those receiving 10-fold less virus (14 versus 6 months) (122). However, in the 120-patient phase IIb part of the study, JX-594, administered to HCC patients who had failed sorafenib therapy, did not significantly increase overall survival compared to individuals receiving standard of care therapy (125). A phase III trial in treatment-naive HCC patients, in combination with sorafenib, is currently recruiting.

Vaccinia virus “double-deleted” virus (VV-DD) combines a TK mutation with a mutation in the VACV growth factor (VGF) gene in a WR strain background (126). VGF^−^ VACV showed pathogenicity in nude mice similar to that of TK^−^ VACV, but when these deletions were combined, the resulting virus showed little ability to replicate outside of tumors (105). VV-DD exhibited a better safety profile compared to wild-type WR in rhesus macaques (109) and increased tumor selectivity compared to TK^−^ mutants *in vitro* (91). It also showed antitumor effects in several tumor-bearing animal models (91, 105, 127, 128). A version of this virus called JX-963, which also encodes GM-CSF, showed improved efficacy over VV-DD in rabbits bearing VX2 liver tumors (91). VV-DD’s oncolytic efficacy might also have been improved by the introduction of a point mutation in A34R, which promotes increased viral spread (129, 130).

Another version of this virus (also called VV-DD (131), VVDD-CDSR, or JX-929), encodes TK and VGF mutations as well as yeast cytosine deaminase and human somatostatin receptor type 2. It has been tested for safety in two phase I clinical trials. Sixteen patients with solid tumors (breast, colon, pancreatic carcinomas, and melanoma) were enrolled in the first trial after having received multiple prior alternative treatments. These patients received intratumoral doses of virus up to 3 × 10^9^ PFU (132). In a second trial, 11 patients with treatment-refractory solid tumors received up to 3 × 10^9^ PFU of virus as a single intravenous dose (133). In both instances the virus was well tolerated, and no maximum tolerable dose was seen. Evidence of viral replication was observed, along with the appearance of virus-specific antibodies, and some very preliminary evidence of efficacy (132, 133). While not used in either study, the virus-encoded somatostatin receptor would permit tracking virus distribution using ^111^In-pentetreotide (128). The encoded yeast cytosine deaminase catalyzes conversion of 5-fluorocytosine to the cytotoxic 5′-FU, enabling combination suicide gene/prodrug/oncolytic virus therapy (127). In an immunocompetent animal model of ovarian carcinomatosis, this combination approach prolonged survival (127).

A Lister-based strain of recombinant VACV called GLV-1h68 or GL-ONC1, combines a TK mutation with mutations in the viral hemagglutinin (*A56R*) and *F14.5L* genes (131, 134). This virus demonstrated reduced virulence in nude mice (134), and oncolytic efficacy in several immune compromised animal models (135–138). Phase I/II trials in patients with head and neck cancer, lung cancer, peritoneal carcinomatosis, and additional solid tumors are currently underway [reviewed in Ref. (139)]. GLV-1h68 has been further modified to express transgenes which permit monitoring virus distribution, or aim to improve oncolytic efficacy (139).

Vaccinia virus TMPK is a functional but non-essential early gene, with viral homologs only found in other orthopoxviruses (140, 141). The catalytically active dimer is structurally related to human TMPK (141, 142). Like human TMPK, the VACV enzyme phosphorylates dTMP, dUMP and a number of dUMP analogs (143). However, unlike human TMPK, VACV TMPK can also phosphorylate dGMP and several different dGMP analogs. This includes O^6^-Me-GMP, which is not a substrate for human TMPK or GMPK (143, 144). This additional activity suggests that VACV TMPK might be a potential target for antiviral drugs. Moreover, given that TMPK functions downstream of TK, it is possible that TMPK mutants would display similar oncolytic properties to TK mutants. Further studies should investigate this possibility.

### VACV RNR Mutants

Vaccinia virus encodes homologs of both the small and large subunits of RNR, products of the *F4L* (145, 146) and *I4L* genes (147), respectively. These virus and human genes are sufficiently similar that some antibodies cross-react with the encoded proteins. VACV R1 and R2 can form functional complexes with each other, and also with cellular R1 or R2. Interestingly, complexes composed of viral R2 and mouse R1 show greater specific activity than cellular complexes, while viral R1 and cellular R2 complexes show reduced activity (148, 149). Like cellular R2, VACV R2 interacts with R1 subunits through its C-terminus, and the residues required for RNR activity are highly conserved (150). Like its cellular counterpart, VACV RNR is also subject to allosteric regulation and is inhibited by hydroxyurea (HU) (145, 146, 148, 151, 152). However, unlike cellular RNR, VACV RNR exhibits little ability to reduce UDP (148). In addition, the virus R2 also bears an N-terminal truncation (150) that deletes sequences corresponding to a region spanning the KEN box motif in the cellular enzyme. The KEN box is recognized by Cdh1-APC, and this leads to its degradation during mitosis (9). The absence of this element in viral R2 would explain the relative stability of this protein during infection (150).

Both the VACV *F4L* and *I4L* genes are non-essential. However, R1 and R2 mutants show different phenotypes, which suggest that the R2 subunit is a more important determinant of viral fitness. There is little difference in growth properties between wild-type VACV and R1 mutants, even in serum-starved (i.e., non-proliferating) cells. In contrast, R2 mutants replicate poorly in serum-starved cells. Although mutations in either gene will enhance virus sensitivity to HU or cidofovir (a nucleoside analog that competes with dCTP) (150), the effect is magnified in R2 mutants. These viruses also greatly differ in virulence. R2 mutants are highly attenuated following intranasal or intravenous administration, while R1 mutants show only mild attenuation (150, 153, 154). However, R1 mutants are attenuated when administered intracranially (153, 155) suggesting that VACV R1 may play some role in neurovirulence (R2 mutants were not tested in these studies). The relative difference in the importance of viral RNR subunits is further illustrated by bioinformatics analysis. R2 homologs are encoded by most poxviruses, while only a subset of genera (*Orthopoxviruses* and *Suipoxviruses*) encode the R1 subunit (150, 156). This suggests that natural selection pressures favor R2 over R1.

Transgene has generated a recombinant WR-based VACV, VV-FCU1, with R1 deleted and the TK locus replaced with a gene encoding yeast cytosine deaminase fused to uracil phosphoribosyl transferase (FCU1) (157). This enzyme fusion converts the prodrug 5-flurocytidine to 5-flurouracil (158), delivering the chemotherapeutic to virus-infected cancer cells. While combining 5-flurocytidine treatment with VV-FCU1 resulted in more sustained control of Renca tumors in immune competent mice, it did not significantly prolong survival over virus alone (157). These studies did not evaluate whether an R1 mutation alone, or combined with other mutations (e.g., TK), affected the oncolytic properties of the virus. Note that VV-FCU1 has unfortunately also been used to designate another FCU1-expressing TK mutant (114). However, the latter is R1^+^ and was generated from the Copenhagen strain of VACV.

R2 deletion reduces the yield of VACV ~1,000-fold compared to wild-type WR, when grown in serum-starved non-transformed cells (e.g., N60 fibroblasts or normal kidney cells) (104). This difference is less apparent in cycling cells (~10-fold), and in some cancer cell lines VACV R2 mutants grow almost as well as the wild-type strain (104, 150). This effect is linked to levels of cellular R2. HeLa cells exhibit elevated amounts of dNTPs (159) and support growth of R2-deficient VACV at levels much like that of wild-type VACV. However, when cellular R2 levels were reduced using siRNA silencing, the yields of R2-deleted VACV mutants were reduced ~10-fold while growth of wild-type VACV was not significantly affected. Furthermore, in BSC-40 or CAPAN-2 cell lines, where VACV R2 mutants grow relatively poorly, virus expression of cellular R2 can compensate for the deficiency (150). The p53-R2 cellular enzyme does not complement viral R2 mutants, perhaps because of the reduced activity relative to R2 (10, 12, 150).

Our laboratories have been investigating whether R2-deficient VACVs (strain WR) can be used as oncolytic agents for treating bladder cancer. These R2-deficient VACVs promote oncolysis in immune compromised mice bearing either subcutaneous or orthotopic human bladder cancer xenografts. Oncolytic activity is also seen in immune competent rats bearing orthotopic bladder tumors (104). In the rat model, long-term complete responses were achieved, and these animals showed evidence of induced antitumor immunity. These R2-deficient VACV strains also appear to be safer (i.e., less virulent) than the TK-deficient strain. Although neither virus induced overt toxicity following administration to the bladder of tumor-bearing immune competent rats, *ex vivo* analysis demonstrated that TK-deficient virus was recoverable from the ovaries, kidneys, and lungs, while the spread of R2-deficient strains was limited to the tumor site. An even greater safety advantage has been seen in immune compromised mice. When TK-deficient virus was administered by either intravenous or intratumoral routes, weight loss and pox lesions were observed. In contrast, R2-deficient viruses did not cause these classic signs of poxvirus virulence.

One can also combine R2 and TK mutations without impairing the antitumor activity of VACV. In fact this combination of mutations (ΔF4LΔJ2R) produces a virus that lacks the virulence of ΔJ2R strains in immune compromised animals while still being able to cure xenografted tumors. Interestingly, fewer virus-specific antibodies were detected in rats infected with R2-deficient VACV relative to TK-deficient virus. This most likely reflects the poorer growth of R2 mutant strains in rat tissues.

### VACV Uracil-N-glycosylase and dUTPase Mutants

Vaccinia virus encodes two proteins that minimize uracil incorporation into virus genomes: a dUTPase (encoded by *F2L*) and a uracil-N-glycosylase (UNG, also designated UDG, encoded by *D4R*). It has been hypothesized that these viral proteins benefit VACV when it infects quiescent cells, which express low levels of these enzymes and have relatively high ratios of dUTP-to-dTTP (160). RNRs can also reduce UDP to dUDP, creating a flux of undesirable dUTP (Figure 1).

The VACV dUTPase is functional (161) and structurally related to mammalian dUTPase (162), but has little influence on viral fitness. Mutants grow to similar levels as wild-type VACV in both dividing and non-dividing cells and exhibit little alteration in virulence (160, 163).

Vaccinia virus UNG has two known functions. It is a functional UNG and catalyzes uracil excision from DNA. However, VACV UNG also has an essential role in DNA replication, distinct from its glycosylase activity. Together with the viral DNA polymerase (E9L) and processivity factor (A20R), VACV UNG forms a virus holoenzyme [reviewed in Ref. (164)]. This role is important because although catalytically inactive UNG mutants are viable, knockouts are not unless cultured in a complementing cell line (165, 166). These catalytically dead mutants show modest reductions in DNA synthesis and viral yields when grown in both dividing and non-dividing cells (160, 165). UNG activity is clearly important *in vivo*, as catalytically inactive UNG VACVs are attenuated (160, 165).

While neither UNG nor dUTPase mutants alone have properties that would be desirable in oncolytic viruses, a virus bearing mutations in both genes might be more useful. A catalytically inactive UNG/dUTPase double mutant was shown to grow selectively in dividing cells, where it grew to similar levels as wild-type VACV. In quiescent cells, this virus grew to 2–3 logs lower levels than wild-type or single mutant viruses (160). This virus might also be more attenuated in normal mouse tissues than a virus bearing mutations in either gene alone (160).

While these observations would suggest that a UNG/dUTPase double mutant may be an oncolytic candidate, two characteristics would argue against this scheme. First, to preserve the essential structural role of UNG in VACV DNA replication, the catalytic domain must be inactivated by point mutation rather than by clean deletion. This creates the possibility of reversion to wild type. Second, given that uracil incorporation into DNA can be mutagenic, a virus that has a reduced ability to repair uracil could be prone to higher mutation rates, resulting in a greater number of defective viral genomes.

## Herpes Simplex Viruses

Members of the *Herpesviridae* infect a wide range of animals. They are large double-stranded DNA viruses that replicate in the nucleus of infected cells, and are characterized by abilities to undergo lytic replication, as well as to establish latency. While most herpes viruses are species-specific, the tissue-tropism of different viruses ranges widely from relatively broad to very narrow—in some cases infecting only a single cell type (167). To date, only human-specific members of the *Alphaherpesvirinae* subfamily have been investigated for their oncolytic properties (Table 3). This includes HSV-1, herpes simplex virus-2 (HSV-2) (168–170), and varicella zoster virus (VZV) (171). Several recombinant HSV-1’s have entered clinical trials, and to date the only FDA-approved oncolytic virus is a modified HSV-1 (Talimogene laherparepvec or T-Vec), which was licensed based on the results of a phase III clinical trial in patients with advanced melanoma (172, 173). T-Vec is deleted in the genes encoding ICP34.5 and ICP47, is engineered to have early expression of *US11*, and encodes the human GM-CSF transgene (174). These alterations in the viral genome do not directly affect nucleotide metabolism but are discussed in more detail below in the context of other oncolytic HSVs.

**Table 3:** Oncolytic herpes viruses with mutations in nucleotide metabolism genes.

Virus	Strain	Viral mutation(s)	Transgene(s)	Status	Reference
Herpes simplex virus-1					
G47Δ^a^	F	R1^−^, ICP34.5^−^, ICP47^−^, *US11* under control of ICP47 promotor	*lacZ*	Completed phase II trial	(204, 205)
NV1020 (R7020)	F	TK^−^ (also disrupts *UL24* expression), 15 kb deletion of joint region (deletes one copy of ICP0, ICP4, LATs, and ICP34.5 genes)	Extra copy of TK placed under ICP4 promoter, HSV-2 glycoproteins inserted in the joint region	Currently in phase II trial	(238–241, 273–275)
rRP450	KOS	R1^−^	CYP2B1	Currently in phase I trial	(189, 191, 195)
hR3	KOS	R1^−^	*lacZ*	Preclinical	(192)
GL207	F	R1^−^, deletion in both copies of ICP34.5 gene	*lacZ*	Preclinical	(197–203, 276–278)
Δ68H-6	17syn+	R1^−^, ICP34.5 beclin1 binding domain deletion	*lacZ*	Preclinical	(209)
*dlsptk^b^*	KOS	TK^−^ (deletion maintains *UL24* expression)		Preclinical	(193, 229, 230)
*KOS-SB*	KOS	TK^−^ (deletion maintains *UL24* expression)		Preclinical	(279)
NV1066	F	TK^−^, deletion of internal repeat region (deleted in one copy of ICP0, ICP4, ICP34.5 genes)	eGFP	Preclinical	(233–237)

**Herpes simplex virus-2**					

FusOn-H2	wt186	Deletion of PK domain in R1, RR domain fused to eGFP and under control of CMV promoter	eGFP	Preclinical	(213, 215)

*^a^G47Δ has been further modified to express transgenes such as IL-18, IL-12, platelet factor 4, or angiotensin (207, 280–282)*.

*^b^The HSV-1 TK gene overlaps with UL24. Depending on the strategy taken to disrupt TK, some HSV-1 TK mutants also have mutations in UL24*.

Herpes simplex virus-1 and HSV-2 can infect both resting and dividing cells and encode R1, R2, TK, dUTPase, and UNG homologs (167). While these proteins can catalyze nucleotide metabolism, some promote reactions not catalyzed by their cellular counterpart, or have additional immune regulatory functions. Because of this, development of viruses bearing mutations in these genes for oncolytic virotherapy requires additional considerations.

### Herpes Virus RNR Mutants

An increase in RNR activity is seen early in HSV-1 infection (175, 176) and HSV-1 encodes homologs of both the R1 and R2 subunits (genes *UL39* and *UL40*, respectively). These viral subunits interact with each other (177, 178) and catalyze the same reactions as cellular RNR. However, HSV-1 RNR activity is regulated differently. Unlike cellular RNR, HSV-1 RNR is subject to ATP inhibition, but is not inhibited by dATP or dTTP (179, 180).

The R1 protein encoded by HSV-1, and other alpha- and beta-herpes viruses, bears an additional ~400 amino acid N-terminal domain incorporating a receptor-interacting protein (RIP) homotypic interaction motif. This domain, while dispensable for R1-R2 binding and RNR activity (177, 181), promotes an interaction between viral R1 and the cellular kinase RIP3 (182). In human cells the interaction between viral R1 and RIP3 disrupts formation of the RIP1/RIP3 necrosome complex, thus preventing activation of antiviral programmed necrosis. In contrast, in mouse cells the viral R1-RIP3 interaction leads to necrosome activation and subsequent cell death (182, 183). This domain also promotes an R1 interaction with eIF4G, which is thought to serve as a scaffold for further interactions with eIF4E, and aid viral mRNA translation (184, 185). This importance of this domain for pathogenesis is confirmed by the observation that murine cytomegalovirus R1 mutants are severely attenuated, despite the fact that MCMV R1 is non-functional as an RNR (186). The fact that R1 serves an additional role in immune regulation and is perhaps needed earlier in the infection cycle than are dNTPs, may rationalize the otherwise puzzling fact that R1 is expressed earlier than R2 (187).

Herpes simplex virus-1 R1 mutants have properties that make them promising oncolytic agents. The R1 mutant hR3 is attenuated in animal models (188, 189) and has also been reported to exhibit defects in reactivation from latency (189). While R1 mutants show reduced replication compared to wild-type HSV-1, they also grow more selectivity in dividing cells. In serum-starved cells, R1 mutants produce 10- to 1,000-fold lower yields of virus and only a third of the DNA synthesis of wild-type virus (153, 190, 191). Cancer cells often bear p16 mutations, which result in increased RR activity (detectable as R2 mRNA), and an R1 HSV-1 mutant was shown to replicate to higher levels in these cells, regardless of whether these cells were dividing or not (192). R1 mutants also show increased sensitivity to the TK substrate prodrugs acyclovir (193) and ganciclovir (194), which may provide additional safety benefits.

An oncolytic HSV-1 (KOS strain) virus called rRP450 was engineered to incorporate a rat *CYP2B1* gene replacing the virus R1 sequence. *CYP2B1* encodes a cytochrome P450 that converts cyclophosphamide into its cytotoxic product. While rRP450 controlled the growth of tumors in a number of model systems, including rat 9L gliosarcomas, human U87 gliomas, murine MC26 liver metastases, and human Rh30 rhabdomyosarcomas, oncolytic efficacy was significantly enhanced when the animals were also given cyclophosphamide (189, 191, 195). This suggests that R1 may serve as an appropriate site for the expression of transgenes in HSV-1. A phase I clinical trial (NCT01071941) in patients with primary liver tumors and metastases is currently underway.

Herpes simplex virus-1 R1 mutations have also been combined with other viral mutations. The HSV ICP34.5 protein serves many functions including preventing host protein shutoff, disrupting the type I interferon response, and inhibiting autophagy [reviewed in Ref. (196)]. It is also a key neurovirulence factor and many oncolytic HSV-1 strains bear ICP34.5 mutations including T-Vec. HSV-1 clone G207 (an F strain) was assembled bearing mutations in R1 and in both copies of the gene encoding ICP34.5. It is attenuated in mice and non-human primates (197, 198) and showed efficacy in many animal tumor models including xenografts of U87 gliomas (199) and F5 meningiomas (200). G207 was well tolerated in a clinical dose-escalation study, where 21 patients with recurrent glioblastomas, gliosarcomas, or anaplastic astrocytomas were enrolled and given a single intratumoral dose of virus (up to 3 × 10^9^ PFU), at 1–5 sites (201). In a subsequent phase Ib trial, patients with recurrent glioblastomas were given two doses of virus totaling 1.15 × 10^9^ PFU, one prior to and one after surgical resection (202). Collectively both trials showed that G207 was well tolerated, replicated *in vivo*, generated neutralizing antibodies, and showed some evidence of antitumor effects in some patients (201, 202). A phase I clinical trial (NCT02457845) in children with progressive or recurrent malignant brain tumors is currently underway (203).

G207 (like T-Vec) has been further modified by deleting the open reading frame encoding ICP47, generating a virus called G47Δ. This deletion has two consequences. The first is to increase immune recognition of infected cells, since the ICP47 protein inhibits MHC-I antigen presentation. Second, this deletion places the nearby *US11* gene under the control of the ICP47 promoter, thus increasing virus yield by preventing premature termination of protein synthesis resulting from loss of ICP34.5 (204, 205). This virus has shown efficacy in a number of tumor models [reviewed in Ref. (206)] including tumor models established using brain or breast cancer stem cells (207, 208). G47Δ has been evaluated in phase I and II clinical trials. The Japanese Ministry of Health, Labor and Welfare gave G47Δ “Sakigake” or breakthrough status in February 2016, based on the positive outcomes of a phase II trial in individuals with recurrent or residual glioblastoma (206).

It has been suggested that deleting genes encoding R1 (*UL39*) and ICP34.5 (*γ_1_34.5*) would compromise the efficacy of these mutant HSV-1 viruses. Kanai et al. studied a combination of a complete *UL39* deletion with partial mutations in *γ_1_34.5* in the 17syn^+^ strain (209). They found that the virus still exhibited the reduced neurovirulence characteristic of strains completely deleted of *γ_1_34.5* while this virus (called Δ68H-6) also exhibited increased efficacy in experimental glioma models (209).

Herpes simplex virus-2 R1 mutants are also being investigated for their oncolytic properties, although from a perspective different from the role the enzyme plays in dNTP biosynthesis. A HSV-2 mutant lacking part of the R1 N-terminal domain (ICP10ΔPK) replicated less efficiently in serum-starved cells and exhibited reduced virulence in animals, despite still being able to complex with R2 and catalyze dNTP synthesis (210, 211). This virus is being tested as a candidate vaccine against genital herpes (212) but can also control the growth of human melanoma xenografts in nude mice (213, 214). A similar version of this virus, called FusOn-H2, also exhibited growth restriction in serum-starved cells and controlled growth of xenografted human MDA-MB-435 melanomas (168) and EC9706 esophageal tumors (215) in nude mice.

Few studies have explored whether HSV-1 R2 mutants might also exhibit oncolytic properties. Unlike the wild-type virus, an HSV-1 R2 mutant (*ts1222*) was unable to replicate in serum-starved BHK21 cells (216). This mutant is also dramatically attenuated in mice (188).

### Herpes Virus TK Mutants

An HSV-1-encoded TK was discovered when it was shown that the virus could productively infect a cell line lacking TK activity (217, 218). HSV-1 TK is encoded by the *UL23* gene and is expressed early in infection (219). Besides phosphorylating pyrimidine nucleosides (dT, dC, dU), HSV-1 TK also exhibits TMPK activity, converting dTMP to dTDP (220, 221). Importantly, HSV-1 TK can also phosphorylate deoxyguanosine and its analogs (222) much more efficiently than cellular TK, and it is this property that has made it a target of many antiviral drugs. The deoxyguanosine analogs acyclovir and ganciclovir are phosphorylated by HSV-1 and other herpes virus TKs. Cellular dGMP kinase and NDP kinases then catalyze further phosphorylation steps, creating the triphosphorylated products. These are then incorporated into DNA and cause chain termination (222–224).

Herpes simplex virus-1 and HSV-2 TK mutants grow to near wild-type levels in cells under normal serum conditions, but growth is reduced 10- to 100-fold in serum-starved cells (225–227). When administered intracranially in BALB/c mice, a TK-deficient HSV-1 strain showed a 100-fold reduction in LD_50_, while a five-log reduction was observed with HSV-2 TK mutants (225). Defective reactivation in infected ganglia was also observed, and these TK defects could be complemented with either human TK1 or TYMS, but not DCK (228, 229). Collectively these data suggest that TK-deficient herpes viruses would selectivity replicate in rapidly dividing cancer cells.

An HSV-1 TK mutant (*dlsptk*) exhibited oncolytic properties in a number of immune compromised animal models. These include human U87 gliomas (230), medullablastomas derived from human Daoy cells, and malignant meningiomas from human M3 cells (231). While showing oncolytic efficacy, HSV-1 TK mutants still exhibit some degree of neurovirulence, with some mice succumbing to virus-related events, presumably encephalitis (230, 232).

NV1066 is a recombinant HSV-1 (F-strain) that combines a *UL23* (TK) deletion (which also disrupts *UL24* expression), with a deletion spanning the internal repeat region. This deletes one copy of each gene encoding ICP0, ICP4, and ICP34.5 (233). NV1066 selectively kills stem-like tumor initiating cells (234), and replicates in animal models of cancers including lymphatic metastases (233), peritoneal carcinomas (235), esophageal adenocarcinomas (236), and a metastatic pleural cancer model derived from A549 cells (237).

NV1020, was originally designed as an HSV-1/-2 vaccine (238). It is similar to NV1066, but it also has an insertion of genes encoding a set of HSV-2 glycoproteins as well as HSV-1 TK (the latter under control of the ICP4 promoter). A number of animal models have been used to show it can control tumor growth including an orthotopic model of bladder cancer (239) and A549-derived tumors established in the pleural cavity of athymic rats (240). NV1020 was studied in a phase I/II trial in patients with advanced metastatic colorectal cancer (241). The virus was well tolerated, with fever and chills being commonly reported. The phase I component of the study did not identify a maximum tolerable dose, and a dose of 10^8^ PFU was used in the phase II arm. Median survival for patients following treatment was 11.8 months.

Despite the promising safety profile and tumor-selective features of HSV TK mutants, TK mutations have not been widely incorporated into oncolytic herpes viruses. The fact that HSV TK is required to bioactivate acyclovir and ganciclovir, providing an important safety net should an adverse response to the virus be observed, may explain why the development of oncolytic herpes viruses has favored retention of a functional TK. On the other hand, while HSV-1 TK mutants may not have gained wide spread use as oncolytic agents, various forms of the HSV-1 TK gene have been used to track virus distribution or used as a suicide gene, in both HSV-1 and other viruses such as Ad (242–244).

### Herpes Virus UNG and dUTPase Mutants

Herpes simplex virus-1 encodes both UNG (*UL2*) (245) and dUTPase (*UL50*) (246) enzymes. Herpes UNG can be coprecipitated with the viral DNA polymerase and the presence of UNG causes the complex to pause upstream of uracil residues in the template strand (247). This could permit DNA repair in a manner linked with DNA synthesis. Unlike VACV, herpes virus UNG-polymerase interactions are not essential for viral viability, as mutant viruses grow nearly normally in dividing NIH 3T3 or BHK C13 cells (245, 248). However, UNG mutants are attenuated in neural tissue, with LD_50_ about 10-fold higher than wild-type virus when administered by intracranial injection and more than 10,000-fold higher when administered peripherally (248).

While no research concerning the oncolytic properties of a virus bearing only an UNG mutation has been reported, a virus bearing mutations in the UNG gene and *γ_1_34.5* has been studied. This doubly mutated herpes virus, called 3616UB, showed efficacy comparable to a virus bearing *γ_1_34.5* mutations in Daoy or SK-M tumor cells xenografts in SCID mice (249). However, 3616UB was more attenuated than either wild type or *γ_1_34.5* mutant viruses after intracranial administration and was also more sensitive to ganciclovir (249).

Herpes simplex virus-1 dUTPase mutants also show decreased neurovirulence and exhibit defects in exit from latency (250, 251). The need for a functional dUTPase appears to be cell type or tissue dependent and is likely affected by the availability of cellular dUTPase. In cycling cells, dUTPase is dispensable for viral replication, with mutant viruses growing to titers as high as wild-type viruses (227, 250). However, in cells with either naturally low dUTPase levels (e.g., SK-N-SH), or where dUTPase levels have been reduced by shRNA-mediated silencing, the yields of dUTPase-deficient HSV-1 are reduced approximately 10-fold. These defects can be complemented by human dUTPase, either overexpressed by the host cell or encoded by the virus (252). The dUTPase also helps maintain the integrity of the virus genome, as dUTPase mutants isolated from brains of infected mice exhibited greater numbers of mutations per genome than wild-type viruses (251).

The effect of the viral dUTPase on neurovirulence is partly regulated by the virus-encoded *US3* kinase, which activates dUTPase by phosphorylating the enzyme at Ser187 (252, 253). Mutating Ser187 attenuates the virus upon intracranial administration but does not affect virulence when administered to the periphery (254). In contrast, a mutant lacking the gene entirely is attenuated via either route of administration. The way virulence is affected by the route of administration could be partially explained by differences in dUTPase activity in different tissues. The fact that HSV-1 dUTPase mutants grow more poorly in cells with low dUTPase levels suggests that these viruses might have superior oncolytic potential. However, the increased mutation rates caused by dUTPase mutations may argue against using these viruses as therapeutics.

## Summary and Conclusion

Large DNA viruses such as VACV and HSV-1 encode a remarkably similar repertoire of nucleotide metabolism genes. Both viruses encode RNR R1 and R2 subunits, a UNG, and dUTPase. Although VACV encodes distinct TK and TMPK enzymes, these functions are consolidated in the TK enzyme of HSV-1. Over the course of virus evolution, the collective activities and substrate specificities of these enzymes may even have played a role in driving drift in the base composition of these viruses. Relative to the human genome, VACV genomes are A + T rich (66%), while HSV-1 genomes are G + C rich (68%). However, not all herpes viruses are G + C rich and not all poxviruses are A + T rich. The more balanced G + C content in VZV (46%) and HHV-8 (53%) can perhaps be explained by the fact that these herpes viruses encode a TYMS in addition to the biosynthetic genes encoded by HSV-1 and HSV-2 (255). Conversely, poxviruses like Orf and molluscum contagiosum lack R2 and TK homologs and in this case oddly exhibit a strikingly *higher* G + C content (~64%) (256, 257).

Vaccinia virus and HSV-1 illustrate an interesting situation that can be productively exploited to assemble cancer-selective viruses. These viruses have followed an evolutionary path that permits the very successful exploitation of a widely encountered biological niche in the form of non-replicating cells. However, in order to efficiently infect non-replicating cells, this infection strategy renders large DNA viruses dependent upon a complement of virus-encoded nucleotide metabolism genes. By mutating these genes, one creates a requirement for the cell to provide the complementing activities and this environment is typically one found in cancer cells. On the other hand, Ads have evolved mechanisms to induce the expression of these dNTP biosynthetic pathways by manipulating systems like those regulated in the cell cycle. Producing cancer specific oncolytic Ads requires an entirely different mutational strategy. However, it should be noted that there is no one particular approach for acquiring dNTPs that has been adopted specifically by any given virus family. For example, as noted above Orf virus does not encode TK or R2 proteins like VACV. Instead it encodes a protein called PACR that stabilizes cellular R2 by preventing its degradation by the APC at the end of G1/S phase (258). All DNA viruses require some source of dNTPs for replication, but evolution has produced different ways of accessing these metabolites and that strategy determines how one goes about manufacturing an oncolytic virus.

Although these observations suggest the possibility that deleting genes encoding TK and RNR might alter the genetic stability of oncolytic viruses, there is little evidence to support this hypothesis. There is some evidence that HSV-1 dUTPase mutations create a mutator phenotype (259), but the situation is less clear with TK mutants. These may exhibit an antimutator phenotype but that phenotype depends upon the host cell type (248, 260). We have passaged these viruses extensively and seen no significant differences in the rate of accumulation of mutations, compared to wild-type virus, as judged by whole genome sequencing.

Further studies are required to shed more light on these questions, especially relating to how virus infection (whether mutant or wild type) perturbs the dNTP pools in infected cells. It is becoming increasingly apparent that nucleotide pools not only directly affect cell and virus replication but also play a key role in modulating immune responses to infection. Space precludes an extensive review of these still emerging investigations, but we would note how SAMHD1 negatively affects the replication of both HSV-1 and VACV in human primary monocyte-derived macrophage (102). This can be attributed to the destruction of dNTPs by the SAMHD1-encoded phosphohydrolase, and the effect of this on VACV replication is exacerbated by virus-encoded TK mutations (102). SAMHD1 is expressed in a variety of cell types (28), including most hematopoietic cells, and this constitutive activity is generally resistant to signaling by a diversity of proinflammatory cytokines (261). Given this situation, one is left to speculate how newly replicated viruses might affect the lymphocytes recruited to sites of infection, and whether deleting the virus genes that are needed to support nucleotide metabolism would protect these newly recruited immune cells from secondary infections. This could perhaps enhance antiviral responses in normal infections and antitumor responses where oncolytic viruses are employed.

## Author Contributions

CI researched and wrote the manuscript. MH and DE provided guidance in manuscript preparation and subsequent editorial input. All authors agreed to be collectively responsible for the work.

## Conflict of Interest Statement

DE has been awarded US patents as a coinventor of related oncolytic virus technologies and is a coowner of Prophysis Inc., which retains an interest in the licensing rights for these technologies. His coauthors otherwise declare that the research was conducted in the absence of any commercial or financial relationships that could be construed as a potential conflict of interest.
